# Deep learning prediction of esophageal squamous cell carcinoma invasion depth from arterial phase enhanced CT images: a binary classification approach

**DOI:** 10.1186/s12911-023-02386-y

**Published:** 2024-01-02

**Authors:** Xiaoli Wu, Hao Wu, Shouliang Miao, Guoquan Cao, Huang Su, Jie Pan, Yilun Xu

**Affiliations:** 1 Department of Gastroenterology, The First Affiliated Hospital of Wenzhou Medical University, Wenzhou, Zhejiang China; 2 Department of Radiology, The First Affiliated Hospital of Wenzhou Medical University, Wenzhou, Zhejiang China; 3 Department of Gastroenterology, Wenzhou Central Hospital, Wenzhou, Zhejiang China

**Keywords:** Esophageal squamous cell carcinoma, Deep learning, Computed tomography

## Abstract

**Background:**

Precise prediction of esophageal squamous cell carcinoma (ESCC) invasion depth is crucial not only for optimizing treatment plans but also for reducing the need for invasive procedures, consequently lowering complications and costs. Despite this, current techniques, which can be invasive and costly, struggle with achieving the necessary precision, highlighting a pressing need for more effective, non-invasive alternatives.

**Method:**

We developed ResoLSTM-Depth, a deep learning model to distinguish ESCC stages T1-T2 from T3-T4. It integrates ResNet-18 and Long Short-Term Memory (LSTM) networks, leveraging their strengths in spatial and sequential data processing. This method uses arterial phase CT scans from ESCC patients. The dataset was meticulously segmented by an experienced radiologist for effective training and validation.

**Results:**

Upon performing five-fold cross-validation, the ResoLSTM-Depth model exhibited commendable performance with an accuracy of 0.857, an AUC of 0.901, a sensitivity of 0.884, and a specificity of 0.828. These results were superior to the ResNet-18 model alone, where the average accuracy is 0.824 and the AUC is 0.879. Attention maps further highlighted influential features for depth prediction, enhancing model interpretability.

**Conclusion:**

ResoLSTM-Depth is a promising tool for ESCC invasion depth prediction. It offers potential for improvement in the staging and therapeutic planning of ESCC.

**Supplementary Information:**

The online version contains supplementary material available at 10.1186/s12911-023-02386-y.

## Introduction

Esophageal cancer (EC) is the sixth leading cause of cancer-related deaths worldwide, which represents a significant global health burden [1]. The disease can be categorized by two main histological subtypes: esophageal adenocarcinoma and esophageal squamous cell carcinoma (ESCC), of which ESCC is the most common subtype, particularly in South-Eastern and Central Asia (79% of the total global ESCC cases) [2, 3]. Unfortunately, the prognosis of ESCC is generally poor, with a five-year survival rate of around 10–30% in most countries, due to the late diagnosis and the aggressive nature of ESCC [4, 5].

A crucial aspect of managing ESCC lies in the accurate assessment of tumor (T) invasion depth, as this determines the staging of the disease and subsequently guides the choice of treatment, which can range from endoscopic resection to esophagectomy and neoadjuvant chemoradiotherapy [6]. The depth of tumor invasion is also an important prognostic factor for ESCC and significantly correlates with the risk of lymph nodal metastasis [7, 8]. Whether the tumor has penetrated beyond T2 (invading into T3 and above) is of utmost importance, as undergoing neoadjuvant chemoradiotherapy prior to surgery has been shown to provide greater benefits for most patients with advanced stage (T3-T4) [9, 10].

Endoscopic ultrasound (EUS), computed tomography (CT), and positron emission tomography (PET) have traditionally been widely used for estimating the T stage in ESCC [11]. EUS is a standard but invasive method for T staging, carrying potential risks of bleeding, infection, and perforation. Moreover, its clinical application is limited by tumor obstruction in around 30% of cases [12, 13]. PET-CT is effective in identifying regions with elevated metabolic activity, demonstrating reasonable sensitivity and specificity for detecting distant metastases. However, it is an expensive modality and has limitations in accurately assessing the local tumor invasion depth due to its limited spatial resolution [14]. Compared to other methods, CT is a non-invasive, widely accessible tool commonly used in most institutions, particularly for patients with lumen stenosis. However, due to the limited contrast resolution of the esophageal wall and the varied growth patterns of esophageal cancer, accurately determining the depth of invasion and distinguishing different histologic layers on CT can be challenging, often surpassing the capabilities of visual inspection alone [15].

Recently, the emergence of artificial intelligence (AI), particularly deep learning, has revolutionized various fields, including medical imaging [16]. Deep learning [17], a subset of AI, mimics the workings of the human brain in processing data for decision-making. It consists of various architectures, among which Convolutional Neural Networks (CNN) and Long Short-Term Memory (LSTM) networks are particularly promising [18, 19]. CNN, a class of deep neural networks, is most applied to analyzing visual images, while LSTM, a type of recurrent neural network, excels at learning from sequences of data [20, 21]. With the aid of these advanced methods, the identification of subtle patterns and features in the images becomes possible, which may not be easily discernible to the human eye. In various areas, such as non-invasive prediction of gene mutations in lung cancer [22], staging liver fibrosis [23], and early diagnosis of upper gastrointestinal cancers [24], deep learning has shown encouraging diagnostic accuracy and efficiency.

As of now, there is a notable absence of deep learning studies specifically focused on predicting the depth of ESCC using CT imaging, highlighting a research gap in this area. Therefore, this study aims to develop a novel deep learning model, integrating CNN and LSTM frameworks, for predicting the depth of tumor invasion in ESCC using arterial phase enhanced CT images. Specifically, the model will perform a binary classification to determine whether the tumor has breached the muscularis layer (T2) or not, therefore predicting between early (T1, T2) and advanced stages (T3, T4) of the disease.

## Materials and methods

### Study Population

**Fig. 1:**
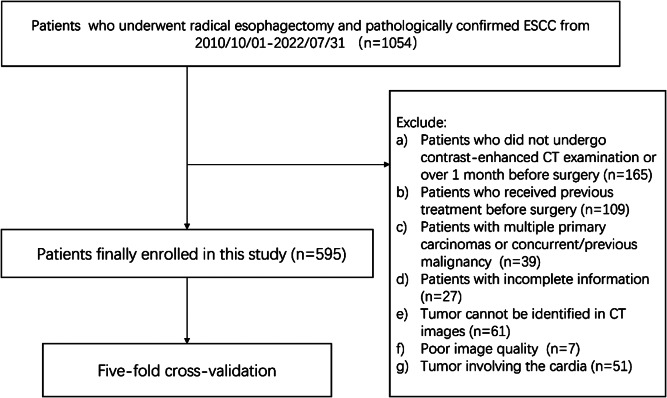
Flow chart representing the study process

The Ethics Committee of the First Affiliated Hospital of Wenzhou Medical University approved this retrospective study and waived the need to obtain informed consent from the patients (ethical code: KY2023-R087). We retrospectively reviewed the clinical records of EC patients who were admitted to the First Affiliated Hospital of Wenzhou Medical University between October 2010 and July 2022. The inclusion criteria were as follows: (1) Underwent radical esophagectomy and pathologically confirmed ESCC. (2) Patients who underwent standard contrast-enhanced CT examinations within 1 month before surgery. (3) Complete clinicopathological information was available. Exclusion criteria included: (1) Patients who received prior treatment such as radiotherapy, chemotherapy, concurrent radio chemotherapy, or esophageal stent placement before surgery. (2) Patients who presented with multiple primary carcinomas or with a concurrent/previous malignancy. (3) The tumor cannot be identified in CT images (too small or too superficial). (4) Clinical information was incomplete. (5) Poor image quality. (6) Tumor involving the cardia. Finally, a total of 595 patients were enrolled (Fig. 1).

### Tumor staging

T staging was performed according to the postoperative pathological examination and the American Joint Committee on Cancer TNM Staging System Manual, 8th Edition [25].

T1 is cancer that invades the lamina propria, muscularis mucosae, or submucosa and is subcategorized into T1a (cancer that invades the lamina propria or muscularis mucosae) and T1b (cancer that invades the submucosa); T2 is cancer that invades the muscularis propria; T3 is cancer that invades the adventitia; T4 is cancer that invades the local structures and is subcategorized as T4a (cancer that invades adjacent structures such as the pleura, pericardium, azygos vein, diaphragm, or peritoneum) and T4b (cancer that invades the major adjacent structures, such as the aorta, vertebral body, or trachea).

### Data Acquisition and Preparation

Patients underwent contrast-enhanced imaging on either a 64-slice CT scanner (GE Healthcare, Milwaukee, Wisconsin) or a 60-slice CT scanner (UNITED IMAGING, Shanghai, China). The CT settings were: 120 KV voltage, automatic mA ranging from 50 to 400, a rotation time of 0.8s, a 512 × 512-pixel matrix, a scan layer thickness of 5 mm, and a scanning direction from head to foot. Contrast-enhanced CT during the arterial phase initiated 25–30 s post-intravenous administration of the iodinated contrast material (Omnipause 350, GE Healthcare, Milwaukee, Wisconsin) at a 3.0 to 3.5 mL/s flow rate using a pump injector (Ulrich CT motion, Ulrich Medical, Ulm, Germany), followed by a 30 ml saline flush. Eligible CT images, stored as Digital Imaging and Communications in Medicine files, were retrieved from the Picture Archiving and Communication Systems at the First Affiliated Hospital of Wenzhou Medical University. The arterial phase was selected for image segmentation to optimally represent esophageal tumors [26]. A radiologist with 7 years’ experience in esophageal imaging, who was unaware of the clinicopathological data but knew that the patients had ESCC, independently determined the region of interest (ROI). The ROI encompassed the entire tumor area, inclusive of the tumor margin. The bounding rectangle’s side length was approximately 1.0-1.2 times the tumor’s diameter. Supplementary Fig. 1 illustrates how a portion of the entire CT image was designated as the ROI. The data management for images used in this study, encompassing data storage, categorization, and annotation, was carried out through the Tencent AIMIS Open Platform.

### Model

In the current study, we put forth the application of a novel deep learning model named ResoLSTM-Depth for the prediction of invasion depth in ESCC. The ResoLSTM-Depth model leverages the synergistic capabilities of CNNs and LSTM networks to efficiently process and learn from medical imaging data.

The CNN component of our model is constructed based on the ResNet-18 architecture [27]. The ResNet-18 structure includes several “Basic Blocks”, each of which contains two 3 × 3 kernel size convolutional layers followed by batch normalization and a rectified linear unit activation function. Each Basic Block is equipped with a skip connection, which enables the direct flow of gradients through the network. This design counters the vanishing gradients problem, enables effective learning from data, and offers a shortcut for performance continuity across layers.

Exploiting the CNN output, a two-layer LSTM network with 128 hidden units processes the sequence of feature maps. LSTM is equipped with memory cells and gate mechanisms that adeptly handle long-term dependencies within data, mitigating traditional recurrent neural network (RNN) gradient issues. Once processed, this output is relayed to a fully connected layer that finalizes the classification task, providing a robust prediction output.

The rectangular ROI of CT images underwent several preprocessing steps prior to their introduction to the CNN. This included resizing the images to a 224 × 224 resolution, restricting the Hounsfield Units to a specific range of [-145, 225], and implementing a minimum-maximum normalization process to scale the image intensities to a range between 0 and 1.

During training, we applied random rotations and flips to the images as a method of data augmentation. For the computation of loss, we leveraged the cross-entropy function, and the Adam optimizer was utilized for the refreshing of network parameters. Utilizing smaller batch sizes can induce noise in model weight adjustments, possibly causing model divergence or suboptimal results. However, larger batch sizes can reduce the model’s effectiveness in real-world applications. Therefore, considering our GPU’s memory capacity, we selected a batch size of 8. Overfitting is likely to occur if the learning rate (LR) is excessively small, while the training process can diverge if the LR is overly large. Instead of keeping the LR static, it should vary within a certain range. Accordingly, in our study, we initialized the LR at 1e-6 and reduced it by 10% every 5 epochs until it fell below 1e-7. The models underwent training for a total of 150 epochs.

**Fig. 2:**
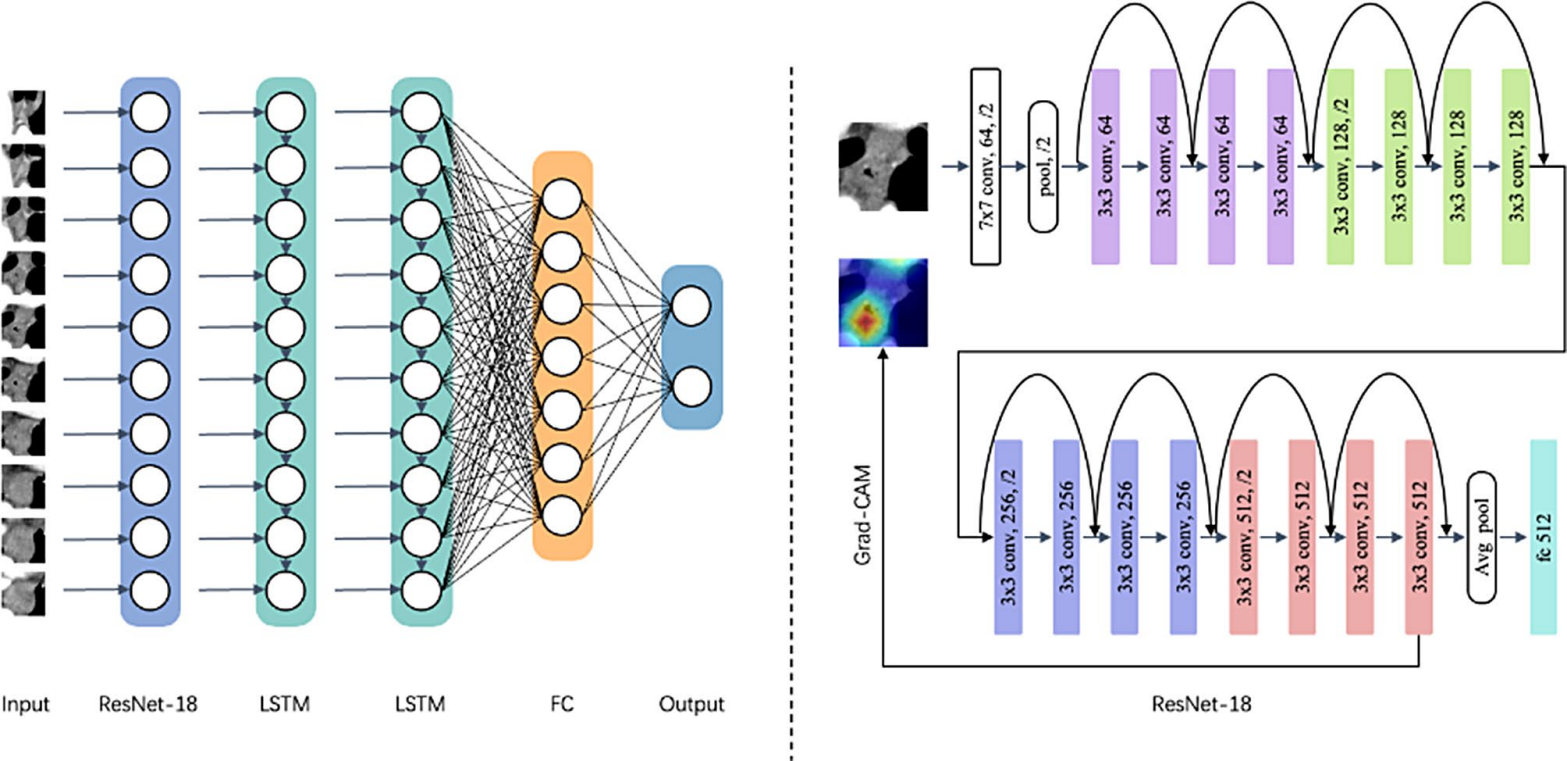
Schematic diagram of the ResoLSTM-Depth deep learning framework

Deep learning networks possess the capability to distinguish images for classification purposes, but pinpointing the exact process of this determination remains challenging. Gradient-weighted Class Activation Mapping (Grad-CAM), introduced by Selvaraju et al. [28], employs deep layer activations weighted by the gradient for transparency and interpretability of CNN-based models. Grad-CAM generates attention maps that visualize critical areas involved in the decision-making process. Applied to the fourth layer of ResNet-18, these attention maps can help highlight the key features associated with the depth of ESCC tumor invasion, thereby enhancing the accuracy of our prediction model. The relevant code is openly accessible at https://github.com/jacobgil/pytorch-grad-cam. Figure 2 illustrates the intricacies of the network structure we designed. The detailed explanation for each network component is presented in the supplementary materials.

All experiments were conducted on a workstation running a 64-bit Windows 11 operating system equipped with 32 GB of memory and an NVIDIA GeForce GTX 2070s GPU. The processes of data normalization and ROI generation were executed in Python (version 3.9). The procedures of data augmentation, training, and validation for all the models were developed using the MONAI (version 1.2) library, with PyTorch (version 1.12.1 with CUDA version 11.3) as the backend.

### Statistic

Our study design involved the division of the dataset into an 80% training set and a 20% validation set. A robust five-fold cross-validation was implemented to maintain the reliability and stability of the model. The validation set was employed to tune the hyperparameters, with the selection criterion having the highest accuracy achieved on this validation set. We gauged the model’s performance using metrics such as accuracy, sensitivity, specificity, F1 score, and the area under the receiver operating characteristic curve. The confusion matrix is used to objectively assess the model’s performance and understand the true positive and misclassification outcomes in the model’s predictions. We applied t-Distributed Stochastic Neighbor Embedding (t-SNE) for dimensionality reduction to illustrate the clustering of T1-T2 versus T3-T4 ESCC stages, the results of which are depicted in the t-SNE plot. These measures provided a comprehensive assessment of the model’s capability to correctly predict the depth of ESCC tumor invasion.

## Result

In this study, a total of 595 cases of esophageal squamous cell carcinoma were included, comprising 141 cases of T1, 151 of T2, 300 of T3, and 3 of T4 stages. Patients had a median age of 68 years, with an interquartile range spanning from 63 to 75. Of these, 525 were male and 70 were female.

**Fig. 3:**
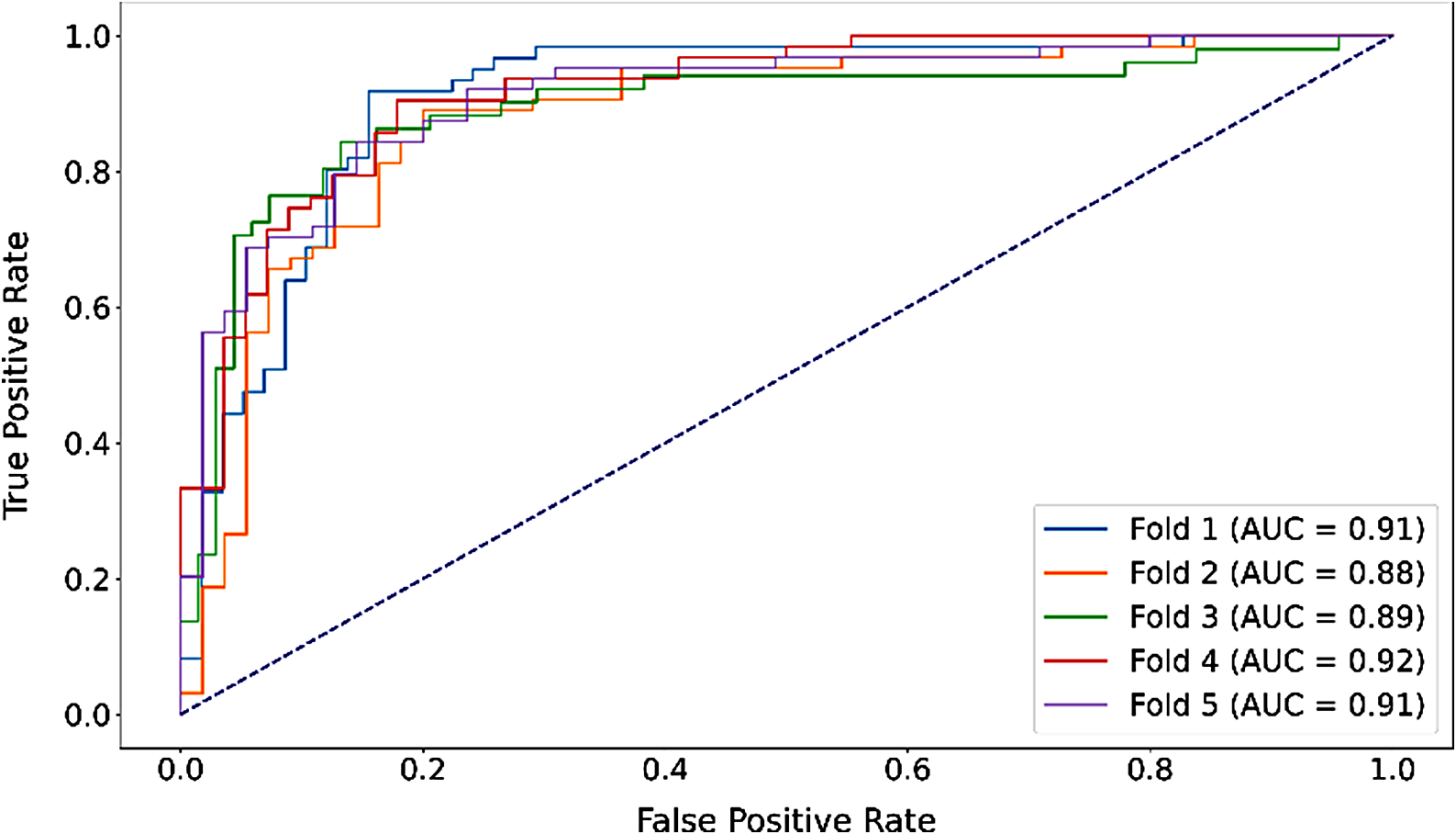
ROC curves from five-fold cross-validation using the ResoLSTM-Depth model

We developed a deep learning model, ResoLSTM-Depth, for predicting the invasion depth of the tumors. The performance of the model was evaluated using five-fold cross-validation with an 80:20 split for training and validation sets, respectively. Across the five iterations, the model achieved the following results: In the first fold, the model obtained an accuracy of 0.882, an area under the curve (AUC) of 0.910, a sensitivity of 0.918, a specificity of 0.845, and an F1 score of 0.889. In the second fold, the performance metrics were 0.840, 0.879, 0.891, 0.782, and 0.857 for accuracy, AUC, sensitivity, specificity, and F1 score, respectively. In the third fold, the model achieved an accuracy of 0.849, an AUC of 0.892, sensitivity of 0.863, specificity of 0.838, and an F1 score of 0.830. For the fourth fold, the model’s accuracy, AUC, sensitivity, specificity, and F1 score were 0.866, 0.916, 0.905, 0.821, and 0.877, respectively. In the final fold, the model yielded an accuracy of 0.849, an AUC of 0.910, a sensitivity of 0.844, a specificity of 0.855, and an F1 score of 0.857. The mean performance of the ResoLSTM-Depth model across all five iterations was characterized by an accuracy of 0.857, an AUC of 0.901, a sensitivity of 0.884, a specificity of 0.828, and an average F1 score of 0.862 (Table 1; Fig. 3). The results of the confusion matrix are displayed in Supplementary Fig. 2. In Supplementary Table 1, the results of using the ResNet-18 model alone to predict in a five-fold cross-validation are presented. The average accuracy is 0.824, and the AUC is 0.879.

**Table 1:** Performance metrics of ResoLSTM-Depth model in five-fold cross-validation

Fold	Accuracy	AUC	Sensitivity	Specificity	F1 score
1	0.882	0.910	0.918	0.845	0.889
2	0.840	0.879	0.891	0.782	0.857
3	0.849	0.892	0.863	0.838	0.830
4	0.866	0.916	0.905	0.821	0.877
5	0.849	0.910	0.844	0.855	0.857
Average	0.857	0.901	0.884	0.828	0.862

The t-SNE analysis provided a compelling visualization of our dataset, distinctly grouping the T1-T2 stages separately from the T3-T4 stages. This clear demarcation in the t-SNE plot reflects the robustness of our model in discriminating between early and advanced ESCC stages. The defined clustering underscores the model’s capacity to discern critical image features necessary for accurate stage classification. These findings are visually represented in supplementary Fig. 3.

Attention maps visualize areas within images that are important for the model’s predictions. In the context of ESCC invasion depth prediction, areas intensely colored (redder) carry higher weights, denoting regions with the most significant features for determining invasion depth. Overlaying these maps onto actual CT images provides a color-coded guide correlating model focus with tumor features.

**Fig. 4:**
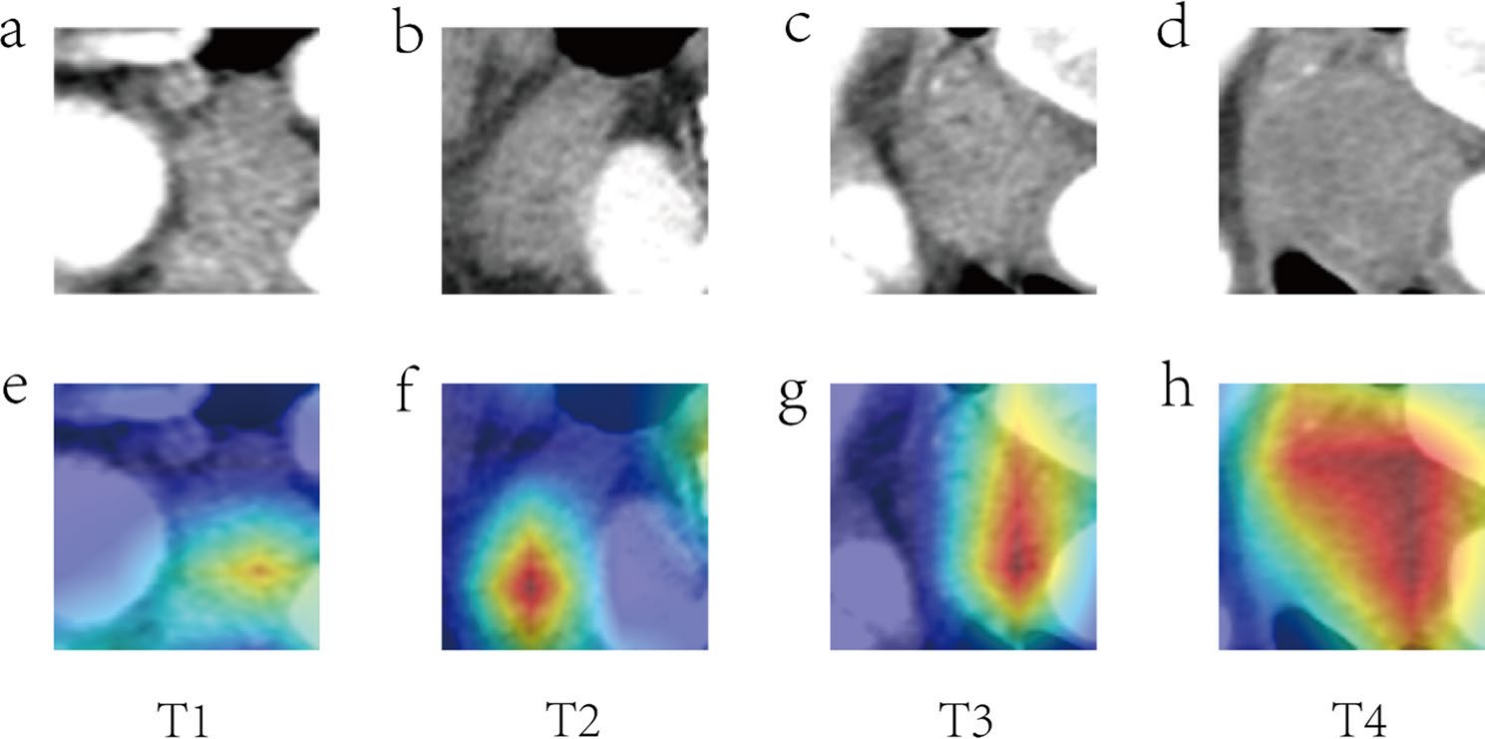
Contrast-enhanced CT images (**a**-**d**) for T1-T4 stages with matching Grad-CAM maps (**e**-**h**), showcasing accurate model predictions across stages

**Fig. 5:**
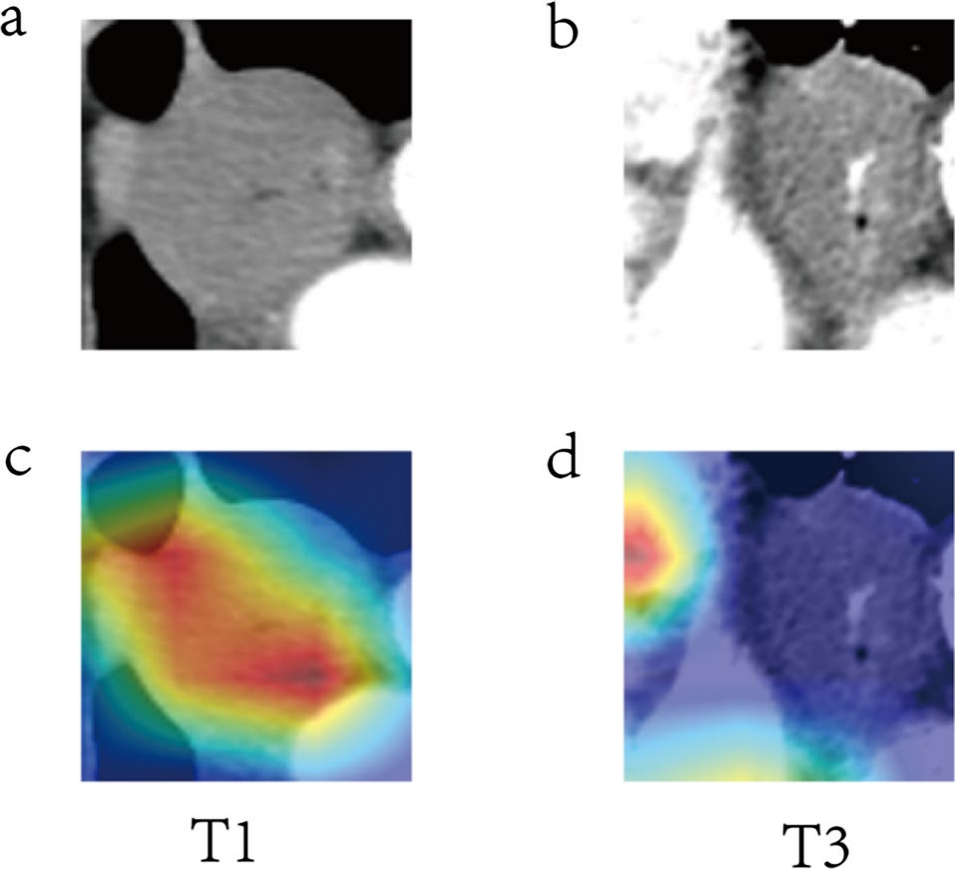
Contrast-enhanced CT examples (**a**, **b**) with corresponding Grad-CAM maps (**c**, **d**) Displaying model mispredictions, with (**a**, **c**) Illustrating inaccuracies in invasion depth and (**b**, **d**) in tumor localization

Figure 4 displays cases where the model accurately predicted tumor invasion depth, exhibiting varying attention maps from T1 to T4 stages. Conversely, Fig. 5 showcases instances of model prediction inaccuracies. The color distribution in the attention maps suggests that Fig. (5a, 5c) corresponds to a prediction error concerning invasion depth, while Fig. (5b, 5d) signifies an error in pinpointing the tumor’s location.

## Discussion

Our study focused on developing a model capable of accurately predicting the invasion depth of ESCC, specifically determining if the tumor had breached the T2 layer. To achieve this, we employed deep learning techniques and designed a model that integrated CNN and LSTM networks. This model exhibited excellent performance with an accuracy of 0.857 and an AUC of 0.901, outperforming the results of the ResNet-18 model alone in this critical prediction. During the generation of predicted results, we employed GRAD-CAM to generate an attention map. The proposed method has important clinical implications. It provides an accurate non-invasive tool for assessing tumor invasion depth in ESCC, aiding in staging, treatment decisions, and improving patient prognosis. This can reduce the need for invasive procedures, lowering complications and costs. The use of GRAD-CAM enhances transparency, trust, and interpretability of the model, facilitating model improvement and potentially assisting in personalized treatment planning.

The assessment of the T stage in esophageal cancer using CT scans relies on various radiological features such as tumor wall thickness, the presence of stenosis, and the morphology of the outer border of the esophageal wall, among others [29]. However, this process has limited applicability in clinical practice and is associated with low accuracy due to its dependence on the expertise and subjective interpretation of radiologists. Wang et al. [30] conducted a study on 1102 patients with ESCC and found that using esophageal wall thickness on CT images had limited accuracy (60.29%) in predicting the invading depth from T1 to T4. Similarly, Yu et al. [31] utilized ultrasonic gastroscopy to measure muscularis propria thickness and muscularis propria + mucosa thickness for determining T2 and T3 patients but achieved a limited accuracy of 68.8%. While tumor thickness can provide some tumor information, it may present one-sided and superficial perspectives. For example, thickening of the esophageal wall may also be caused by inflammation and edema.

Radiomics is a technology that extracts quantitative features from medical imaging data and uses machine learning algorithms to construct prediction models [32]. Wu et al. [33] conducted a study on ESCC patients, analyzing a sample of 154 individuals. They constructed a radiomic model by extracting 10 significant features from contrast-enhanced CT images, aiming to identify stages I-II and III-IV ESCC before treatment. The validation cohort yielded an AUC value of 0.762. Similarly, Yang et al. [15] conducted a study with 116 ESCC patients, utilizing contrast-enhanced CT to extract radiomic signatures. They constructed a radiomic model based on 2 features extracted from three-dimensional tumor regions, achieving an AUC of 0.86 in differentiating T1-T2 from T3-T4 ESCC patients. It is important to note that both studies had relatively small sample sizes, and Wu et al. [33] extracted 10 features, while Yang et al. [15] utilized only 2 features for their radiomic models.

In contrast to radiomics, deep learning can automatically learn and extract features from raw data, eliminating the need for hand-crafted feature engineering, which is required in radiomics [34]. Furthermore, deep learning models can handle a large amount of data and extract complex patterns, which can lead to more accurate and robust predictions. The models can be continually updated and improved as they learn from new data. Our study presents a pioneering deep learning framework, ingeniously marrying the capabilities of CNN with LSTM networks. This unified architecture not only capitalizes on the innate strengths of CNN for robust image feature extraction but also harnesses LSTM’s prowess in deciphering temporal sequences, which traditional CNNs might overlook. In the realm of medical imaging, where precise delineation of tumor boundaries and the understanding of their evolution over sequential scans are paramount, the CNN excels at delineating tumor morphology and localizing critical features within individual image slices. Meanwhile, LSTM layers complement this by interpreting how these features evolve across subsequent slices, a key indicator of tumor invasion progression. The LSTM’s unique memory cells are adept at recognizing and learning from the patterns in data sequences, enabling the identification of tumor invasion depth with greater temporal coherence. By integrating CNN and LSTM, our model seeks to effectively navigate the complexities of medical imaging data. It not only identifies crucial spatial features within image slices but also traces their trajectory across the series of scans, providing a holistic view of the tumor’s architecture and behavior. This dual capacity ensures that our model is particularly attuned to tasks necessitating a comprehensive understanding of the tumor’s three-dimensional structure, underpinning both spatial and temporal data.

To the best of our knowledge, this study represents the first attempt to utilize a deep learning model specifically tailored for distinguishing between T1-T2 and T3-T4 ESCC, demonstrating satisfactory performance. To mitigate the risk of overfitting, we employed a five-fold cross-validation approach, which strikes a balanced compromise between bias and variance. This methodology is particularly advantageous when working with limited datasets, where each data point is highly valuable. By employing five-fold cross-validation, we ensured a reliable evaluation of our CNN + LSTM model’s performance, thereby enhancing the robustness of our findings. While our model achieves the highest AUC and accuracy in comparison to prior studies, it’s not entirely reasonable to draw definitive conclusions on which predictive method is superior based solely on these metrics, due to the sample size, demographic characteristics, modalities, etc. Instead, our model predominantly illustrates the potential and utility of AI in normal clinical practice.

There are some limitations in our study. First, we used thick-slice (5 mm) CT images rather than thin-slice images for the deep learning model training. Compared to thick slice CT, thin-slice CT may reflect more continuous and comprehensive tumor information. Secondly, this study is a single center retrospective study, and although we have included a larger sample of ESCC patients, the reliability of the model still requires external validation. Finally, we only focus on the T stage in this study, the N stage and distant metastases can be explored in future studies to obtain a complete TNM stage for ESCC patients.

## Conclusion

In conclusion, we constructed a deep learning model named ResoLSTM-Depth, which exhibits excellent discrimination capability in differentiating T1-T2 from T3-T4 ESCC. It may serve as a convenient tool for clinicians to predict ESCC invasion depth and guide individualized treatment selection for ESCC patients, although the reliability of the model still requires further clinical validation.

### Electronic supplementary material

Below is the link to the electronic supplementary material.

**Supplementary Material 1**: The supplementary materials include several figures that provide additional insights into the study. These figures encompass various aspects such as the identification of the Region of Interest (ROI) within full CT images, confusion matrices from the ResoLSTM-Depth model during five-fold cross-validation, and a composite t-SNE visualization showing the clustering of different ESCC stages.Additionally, there is a table detailing the performance metrics of the ResNet-18 model across the five-fold cross-validation, and a comprehensive breakdown of the ResoLSTM-Depth model’s components and data flow.These supplementary figures and tables offer a deeper understanding of the methodology and results of the study

## Data Availability

The datasets used and analyzed during the current study are available from the corresponding author on reasonable request.

## Abbreviations

AI: artificial intelligence
AUC: area under the curve
CNN: convolutional neural networks
CT: computed tomography
EC: esophageal cancer
ESCC: esophageal squamous cell carcinoma
EUS: endoscopic ultrasound
Grad-CAM: gradient-weighted Class Activation Mapping
LSTM: long short-term memory
LR: learning rate
PET: positron emission tomography
RNN: recurrent neural network
ROI: region of interest
