# Supplementary material for: Deep learning prediction of esophageal squamous cell carcinoma invasion depth from arterial phase enhanced CT images: a binary classification approach
Source: BMC Med Inform Decis Mak. 2024 Jan 2;24:3. doi: 10.1186/s12911-023-02386-y (PMC10759510; doi:10.1186/s12911-023-02386-y)
Supplement: Supplementary file 1 — Supplementary Material 1: The supplementary materials include several figures that provide additional insights into the study. These figures encompass various aspects such as the identification of the Region of Interest (ROI) within full CT images, confusion matrices from the ResoLSTM-Depth model during five-fold cross-validation, and a composite t-SNE visualization showing the clustering of different ESCC stages.Additionally, there is a table detailing the performance metrics of the ResNet-18 model across the five-fold cross-validation, and a comprehensive breakdown of the ResoLSTM-Depth model’s components and data flow.These supplementary figures and tables offer a deeper understanding of the methodology and results of the study [file 12911_2023_2386_MOESM1_ESM.docx]

Supplementary Figure 1: Illustration of the Designated ROI Within the Full CT Image. The ROI encompassed the entire tumor area, inclusive of the tumor margin. The bounding rectangle’s side length was approximately 1.0–1.2 times the tumor’s diameter in each layer. The final ROI is the combination of each layer's ROI and, thus, can include the whole tumor lesion.


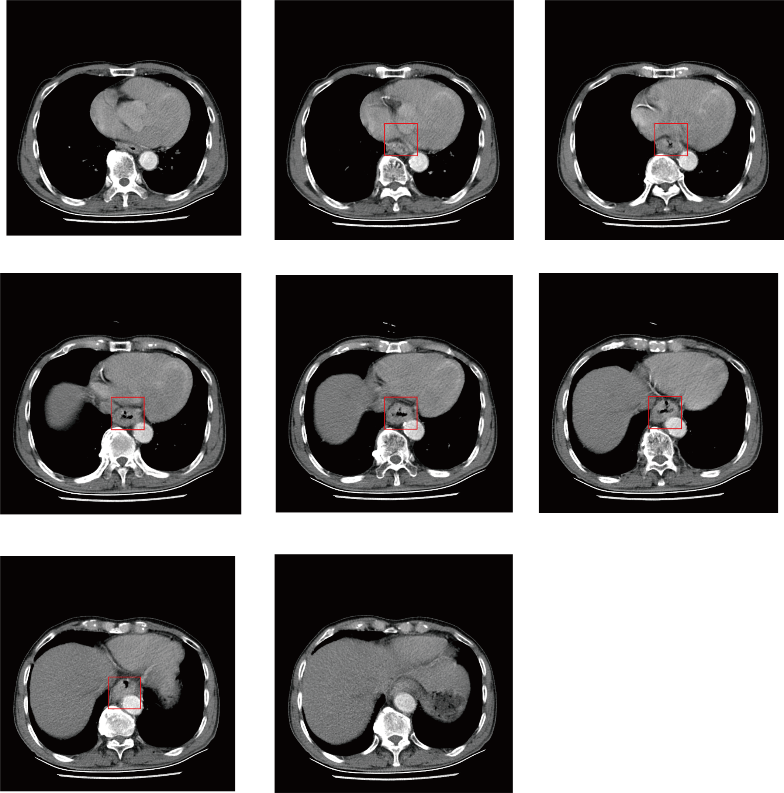


Supplementary Figure 2: Confusion Matrices of ResoLSTM-Depth Model in Five-Fold Cross-Validation


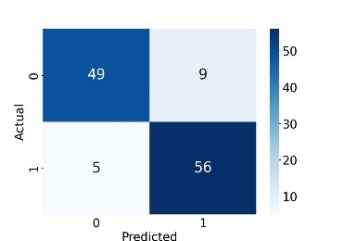

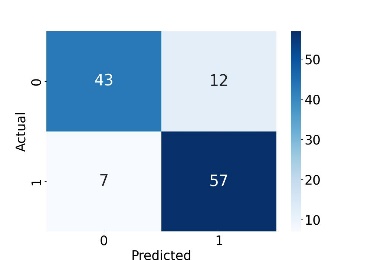

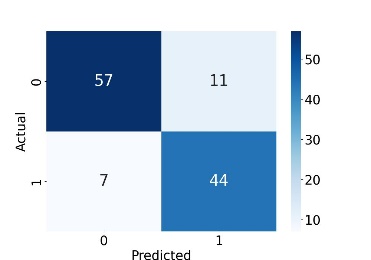

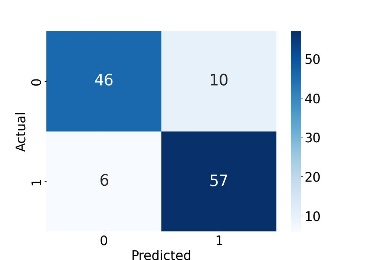

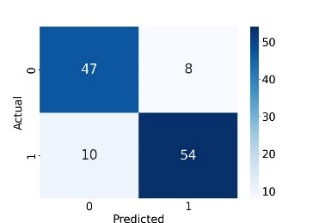


Supplementary Figure 3: Composite t-SNE Visualization of Five-Fold Cross-Validation Results Showing Distinct Clustering of T1-T2 Versus T3-T4 ESCC Stages.


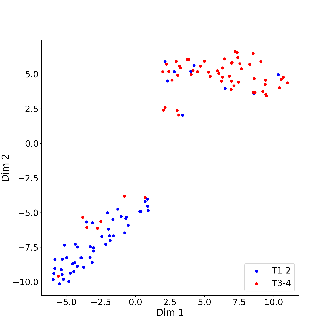

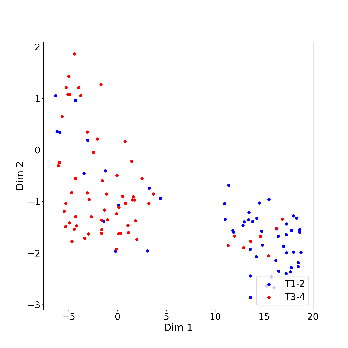

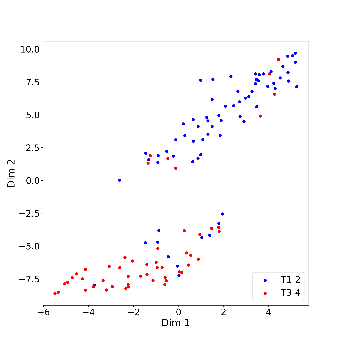


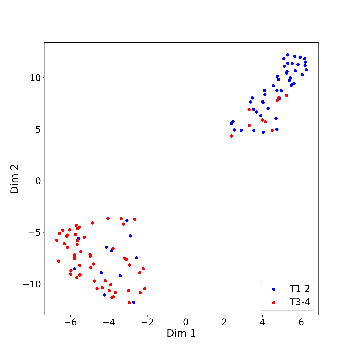

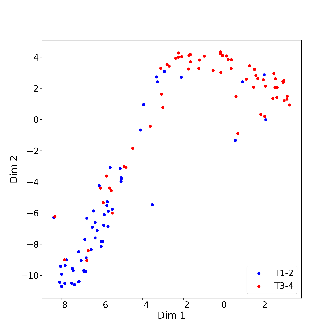


Supplementary Table 1: Performance Metrics of ResNet-18 Model in Five-Fold Cross-Validation

| Fold | Accuracy | AUC | Sensitivity | Specificity |
| --- | --- | --- | --- | --- |
| 1 | 0.849 | 0.890 | 0.875 | 0.831 |
| 2 | 0.790 | 0.862 | 0.828 | 0.746 |
| 3 | 0.824 | 0.888 | 0.877 | 0.759 |
| 4 | 0.832 | 0.901 | 0.800 | 0.878 |
| 5 | 0.824 | 0.856 | 0.821 | 0.825 |
| Average | 0.824 | 0.879 | 0.840 | 0.808 |

**The detailed explanation for each network component**

ResoLSTM-Depth Model Breakdown

Model Overview:

`ResoLSTM-Depth` is an innovative combination of convolutional and recurrent architectures, designed to process sequence data with spatial elements, such as video frames or consecutive medical scans.

1. Preprocessing Convolutional Layer:

Before diving into deep features extraction, the model uses a preliminary convolutional layer. This layer transforms the input data's channel count to match the subsequent network's requirement. Specifically, it prepares the data to have three channels, readying it for the pretrained ResNet that traditionally processes colored images.

2. CNN Component:

The model leverages the renowned CNN, ResNet18, architecture, pretrained on ImageNet. ResNet's strengths lie in its ability to process deep networks without being hindered by the vanishing gradient problem, thanks to its 'skip connections' or residual connections. In this context, ResNet18 acts as the feature extractor.

3. Dimensionality Reduction Layer:

After extracting high-level features via ResNet18, there's a fully connected layer. Its role is to condense these features into a more compact form, thus reducing the feature's dimensions.

4. LSTM Component:

Here's where the sequential aspect of the data is addressed. The LSTM, or Long Short-Term Memory, handles sequence data excellently, retaining essential past information and forgetting the unnecessary. In the model, it accepts the compacted feature representation and processes it through time, capturing the inherent temporal relations in the sequence.

5. Classification Layer:

Finally, there's another fully connected layer that translates the LSTM's output into prediction scores for each class. Depending on the application, this could signify different disease states, various activities, or any classification the model is trained for.

6. Data Flow:

When data enters the network, it undergoes a series of transformations. It's first adapted by the preprocessing convolutional layer, then significant features are extracted using ResNet18, which are then condensed. This condensed representation flows through the LSTM, capturing sequential nuances. Finally, the processed data is classified into one of the pre-defined categories.

This `ResoLSTM-Depth` model ingeniously harnesses the spatial prowess of convolutional networks and the sequential mastery of recurrent networks for enhanced performance on spatial-temporal data.
